# Characteristics of body composition and cardiometabolic risk of Japanese male heavyweight Judo athletes

**DOI:** 10.1186/s40101-016-0092-8

**Published:** 2016-04-06

**Authors:** Hiroko Murata, Satomi Oshima, Suguru Torii, Motoko Taguchi, Mitsuru Higuchi

**Affiliations:** 1 Faculty of Sport Sciences, Waseda University, 2-579-15 Mikajima, Tokorozawa-shi, Saitama, 359-1192 Japan; 2 Graduate school of Sport Sciences, Waseda University, 112 Frontier Reseach Center 135-1 Horinouchi, Tokorozawa-shi, Saitama, 359-1192 Japan

**Keywords:** Visceral fat, Obesity, Metabolically healthy obese, Uric acid

## Abstract

**Background:**

The purpose of this study was to clarify the characteristics of body composition and cardiometabolic risk of Japanese male heavyweight Judo athletes compared with heavyweight athletes of other sports.

**Methods:**

Nineteen heavyweight Judo athletes (mean age, 20.4 ± 1.1 years), as well as 22 heavyweight (mean age, 21.5 ± 0.9 years) and 17 nonheavyweight (mean age, 21.1 ± 0.8 years) American football and Rugby football athletes in Japan participated in this study. Body composition was assessed by using dual-energy X-ray absorptiometry and magnetic resonance imaging. Cardiometabolic risk was evaluated by measuring blood biochemical variables.

**Results:**

Heavyweight Judo athletes had significantly heavier body mass (122.7 ± 13.1 kg vs. 99.0 ± 8.1 kg), higher body fat percentage (27.5 % ± 5.2 % vs. 19.4 % ± 4.7 %), and larger visceral fat cross-sectional area (118 ± 35 cm^2^ vs. 67 ± 24 cm^2^) (*P* < 0.01) compared with heavyweight football players. Although the cardiometabolic risk was higher in heavyweight athletes compared to nonheavyweight athletes, there were no significant differences between heavyweight Judo and heavyweight Football athletes in the blood biochemical variables, except for high concentration of uric acid in heavyweight Judo athletes.

**Conclusions:**

Even though heavyweight Judo athletes had more excess fat mass, especially VF mass, their cardiometabolic risk in terms of blood biochemical parameters was not significantly higher compared with heavyweight athletes of other sports. Therefore, excessive fat accumulation may not necessarily increase cardiometabolic risk for heavyweight Judo athletes.

**Trial registration:**

This trial is registered with the University Hospital Medical Information Network Clinical Trial Registration (UMIN-CTR) UMIN000020564.

## Background

Obesity, especially having visceral fat accumulation, is known to be a major risk factor in cardiovascular disease [1, 2]. Heavyweight athletes, including those who participate in Judo, American football, and Rugby football, often intentionally try to increase their body mass (BM) because it significantly influences their athletic performance [3]. However, it was recently reported that heavyweight athletes have high cardiometabolic risk compared with nonheavyweight athletes, even though they are actively participating in a sport [4–6]. Linemen in the National Football League in the USA have higher body fat percentage compared with players in other positions, and such excess body fat may increase the risk of metabolic syndrome, when they are actively playing [4]. This association is seen not only in Western athletes but also among Asian athletes [7]. Professional Chinese heavyweight athletes in strength sports including Judo and Wrestling are at significantly higher risk of cardiovascular disease compared with other sports or athletes in lower body weight classes of a same sport [7].

The heaviest weight class in Judo, over 100 kg class for male and over 78 kg for female does not have an upper limit in their BM regulation when athletes enter competitions. Therefore, Judo athletes in the heaviest weight class put significant effort toward maintaining or gaining BM. In fact, a previous study showed that heavyweight Judo athletes carry one of the heaviest BM and highest body fat percentage compared with other heavyweight athletes in other sports such as wrestlers and weightlifters [7]. As a result, heavyweight Judo athletes may have higher cardiometabolic risk compared with other heavyweight athletes. However, the previous study did not investigate body composition in detail, such as measuring body fat percentage by using dual-energy X-ray absorptiometry (DXA) and visceral fat (VF) by using magnetic resonance imaging (MRI) in relationship with cardiometabolic risk. If heavyweight Judo athletes indeed have high cardiometabolic risk, such risk may harm their present health. Furthermore, it may increase their risk of cardiovascular disease after they retire from their playing sport [8]. Therefore, the purpose of this study was to clarify the characteristics of body composition and cardiometabolic risk of Japanese male heavyweight Judo athletes compared with heavyweight athletes of other sports. We hypothesized that because heavyweight Judo athletes had higher body fat percentage [7], they expected to have excessive VF mass and increase cardiometabolic risk compared with heavyweight athletes in other sports.

## Methods

### Participants

We recruited 19 collegiate heavyweight Judo athletes from the All Japan University Judo Championships team as the heavyweight Judo group (mean age, 20.4 ± 1.1 years). In 19 heavyweight Judo athletes, 18 athletes were categorized in over 100 kg weight class and one athlete was categorized 100 kg weight class without intentional weight loss. For comparison, 22 heavyweight athletes (BM, >90 kg) were recruited from linemen on an American football team and from forwards position on a Rugby football team as the heavyweight football group (mean age, 21.5 ± 0.9 years). Additionally, 17 nonheavyweight athletes (BM, <90 kg) also were recruited from the previously mentioned American football and Rugby football teams as the nonheavyweight group (mean age, 21.1 ± 0.8 years). These teams belonged to the National Collegiate Athletic Association Division 1 in Japan. All participants in this study were adequately informed about the study by verbal and written descriptions, and each participant provided written informed consent before starting the study. This study was approved by the Ethical Committee of Human Research of Waseda University for use of human subjects in accordance with the Declaration of Helsinki.

### Design and procedure

The present cross-sectional study was designed to compare heavyweight Judo athletes (heavyweight Judo group) and heavyweight football athletes (BM, >90 kg; heavyweight football group). Additionally, in order to emphasize the cardiometabolic risk of heavyweight athletes, “heavyweight group,” which included both heavyweight Judo and heavyweight football athletes, was setup to compare heavyweight and nonheavyweight athletes. Lower weight class Judo athletes were excluded from this study, since they were often involved in an intentional weight loss which may influence their metabolic state. We examined body composition parameters in detail by using DXA and MRI, while cardiometabolic risk was assessed by measuring blood biochemical variables in fasting state. All the measurements of participants were collected in the same laboratory facility in the morning during in-season

### Measurement of body composition parameters

BM was measured to the nearest 0.1 kg by using an electronic scale (UC-321; A&D, Tokyo, Japan) under fasting condition in the morning. Body height was measured to the nearest 0.1 cm by using a stadiometer (YL-65; YAGAMI, Nagoya, Japan) with minimal clothing. Body mass index (BMI) was calculated from the measured BM (kg) and height (m). Body fat percentage was assessed by using DXA (Hologic QDT-4500; Hologic, Waltham, MA, USA). Total fat mass and fat-free mass (FFM) were calculated from BM and body fat percentage. Waist circumference was measured to the nearest 0.1 cm by using an inelastic measuring tape at the midpoint between the inferior coastal margin and the superior border of the iliac crest. VF and subcutaneous fat (SF) cross-sectional areas were measured by using MRI (Signa 1.5T; General Electric Co., Milwaukee, WI, USA). Imaging conditions included a T1-weighted spin-echo and axial-plane sequence with a slice thickness of 10 mm, repetition time of 140 ms, and echo time of 12.3 ms. Cross-sectional images were scanned at the L4-5 level, which was commonly used in previous studies, especially in obese populations [9]. During scanning, subjects were required to hold their breath for approximately 30 s after inhalation to reduce respiratory motion artifacts. The images were transferred to a personal computer in DICOM file format. VF and SF cross-sectional areas were determined using digital image analysis software (Slice-O-Matic 4.3 for Windows; Tomovision, Montreal, Canada). To minimize interobserver variation, all analyses were performed by the same investigator. The coefficient of variation was 2.4 % for VF cross-sectional area and 0.1 % for SF, respectively.

### Collection and analysis of blood samples

Venous blood samples were collected between 7:00 AM and 10:00 AM after at least 12 h of overnight fasting. Aspartate aminotransferase (AST), alanine aminotransferase (ALT), and γ-glutamyl transpeptidase (γ-GTP) were used as indices of liver function. Total cholesterol, high-density lipoprotein cholesterol (HDL-C), and triglycerides (TGs) were analyzed as indices of the lipid profile. Low-density lipoprotein cholesterol (LDL-C) was calculated using the Friedewald equation [10]. Uric acid concentration (UA) was used as the index of uric acid metabolism. Furthermore, plasma glucose, serum insulin, and homeostasis model assessment insulin resistance (HOMA-IR) were assessed as indices of glucose metabolism. HOMA-IR was calculated from plasma glucose and serum insulin concentrations. All blood parameters were analyzed by Mitsubishi Chemical Medience, Inc. (Tokyo, Japan).

### Statistical analyses

All statistical analyses were performed using SPSS version 22.0 (SPSS, Chicago, IL, USA). We examined differences in body composition variables and blood biochemical variables between heavyweight athletes group and nonheavyweight athletes group as well as the heavyweight Judo athletes group and heavyweight football athletes group using the unpaired *t* test. Non-normally distributed variables were log transformed for analysis. Prevalence of each cardiometabolic risk factor was compared between heavyweight Judo, heavyweight football, and nonheavyweight groups using the chi-square test. All measurements were presented as mean ± SD or median (from 25 to 75 % quartile range). The statistical significance level was set at *P* < 0.05.

## Results

### Body composition

The comparison of body composition variables between heavyweight group and nonheavyweight group was presented in Table 1. All body composition parameters were significantly higher in the heavyweight group, except for body height. Table 2 showed the comparison of body composition parameters between heavyweight Judo and heavyweight football groups. The heavyweight Judo group showed significantly higher values in the parameters of fatness compared with the heavyweight football group such as total fat percentage and VF.

**Table 1:** Comparison of body composition variables between heavyweight and nonheavyweight groups

		Heavyweight	Nonheavyweight	*P* value^†^
		(*n* = 41)	(*n* = 17)	
Height (cm)		177.4 ± 5.7	175.6 ± 3.9	0.218
Body mass (kg)^†^		110.0 ± 16.0	78.9 ± 5.0	<0.001
BMI (kg/m^2^)		35.0 ± 4.9	25.6 ± 1.9	<0.001
Total	fat percentage (%)	23.2 ± 6.0	13.0 ± 2.6	<0.001
	fat mass (kg)^†^	26.2 ± 10.1	10.3 ± 2.6	<0.001
	fat free mass (kg)	83.8 ± 8.1	68.5 ± 3.4	<0.001
Waist circumference (cm)		103 ± 10	82 ± 4	<0.001
VF (cm^2^)^†^		91 ± 39	33 ± 14	<0.001
SF (cm^2^)		321 ± 135	94 ± 46	<0.001

Data were expressed as mean ± SD. *BMI* body mass index, *VF* visceral fat area, *SF* subcutaneous fat area; ^*^Log-transformed variables for non-normally distributed variables (body mass, total fat mass, VF) were used. ^†^ *P* value for difference between heavyweight Judo and football groups was assessed by unpaired *t* test. Significant differences, *P* < 0.05

**Table 2:** Comparison of body composition variables between heavyweight Judo and football groups

		Heavyweight (*n* = 41)		*P* value^†^
		Judo (*n* = 19)	Football (*n* = 22)	
Height (cm)		177.2 ± 6.1	177.6 ± 5.6	0.837
Body mass (kg)^*^		122.7 ± 13.1	99.0 ± 8.1	<0.001
BMI (kg/m^2^)		39.1 ± 3.8	31.4 ± 2.3	<0.001
Total fat percentage (%)		27.5 ± 5.2	19.4 ± 3.7	<0.001
	fat mass (kg)^*^	34.1 ± 8.8	19.4 ± 4.7	<0.001
	fat free mass (kg)	88.6 ± 8.0	79.7 ± 5.5	0.005
Waist circumference (cm)		111 ± 7	96 ± 7	<0.001
VF (cm^2^)^*^		118 ± 35	67 ± 24	<0.001
SF (cm^2^)		433 ± 100	223 ± 70	<0.001

Data were expressed as mean ± SD. *BMI* body mass index, *VF* visceral fat area, *SF* subcutaneous fat area; *Log-transformed variables for non-normally distributed variables (body mass, total fat mass, VF) were used. ^†^ *P* value for difference between heavyweight Judo and football groups was assessed by unpaired *t* test. Significant differences, *P* < 0.05

### Cardiometabolic risk

**Fig. 1:**
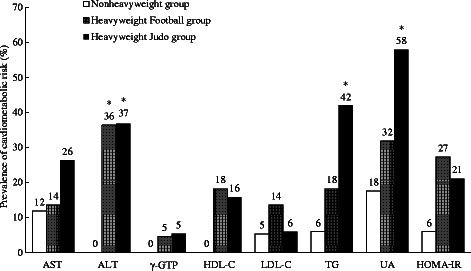
Comparison of prevalence in cardiometabolic risk in terms of blood biochemical parameters. Prevalence of cardiometabolic risk presents the percentage over the referenced normal range of respective parameters. The cut-off referenced normal range were as follows: AST < 40 (U/L), ALT < 45 (U/L), γ-GTP ≧ 80 (U/L), HDL-C < 40 (mg/dL), LDL-C ≦ 140 (mg/dL), TG ≧ 150 (mg/dL), UA > 7.0 (mg/dL), HOMA-IR ≧ 2.5. *Significant differences compared with Nonheavyweight group, *P* < 0.05

The comparison of blood biochemical variables between heavyweight group and nonheavyweight group were presented in Table 3. ALT, γ-GPT, UA, glucose, insulin, and HOMA-IR in heavyweight group were significantly higher than in nonheavyweight group. Table 4 showed the comparison of blood biochemical variables between heavyweight Judo group and heavyweight football. Only UA concentration was significantly higher in the heavyweight Judo group compared with in the heavyweight football group. Figure 1 showed the prevalence of cardiometabolic risk assessed by blood biochemical analysis in all three groups. The prevalence of abnormally high ALT in both the heavyweight Judo and heavyweight football groups was significantly higher than that in the nonheavyweight group. Additionally, the prevalence of abnormally high UA and TG levels in the heavyweight Judo group was significantly higher than those in the nonheavyweight group. The prevalence of other components, such as AST, γ-GTP, HDL-C, LDL-C, and HOMA-IR, were not significantly different between the three groups.

**Table 3:** Comparison of blood biochemical parameters between heavyweight and nonheavyweight groups

	Heavyweight	Nonheavyweight	*P* value^‡^	Normal range
	(*n* = 41)	(*n* = 17)		
AST* (U/L)	28 (24–38)	24 (20–30)	0.053	10–40
ALT* (U/L)	38 (29–49)	20 (15–27)	<0.001	5–45
γ-GTP* (U/L)	32 (26–44)	23 (15–26)	<0.001	<80
Total-C (mg/dL)	179 ± 32	179 ± 31	0.966	120–219
HDL-C (mg/dL)	48 ± 8	51 ± 6	0.220	40–85
LDL-C^†^ (mg/dL)	105 ± 30	109 ± 24	0.581	≦140
TG* (mg/dL)	112 (75–165)	80 (66–110)	0.086	30–149
UA (mg/dL)	6.9 ± 1.5	6.2 ± 1.0	0.048	3.8–7.0
FPG* (mg/dL)	85 (82–91)	80 (78–84)	0.012	70–109
Insulin* (μIU/mL)	8.4 (5.7–11.3)	5.3 (4.4–6.5)	0.011	1.7–10.4
HOMA-IR*	1.72 (1.13–2.52)	1.02 (0.91–1.36)	0.008	<2.50

Data were expressed as mean ± SD or median (inter quartile range). *AST* aspartate aminotransferase, *ALT* alanine aminotransferase, *γ-GTP* γ-glutamyl transpeptidase, *Total-C* total cholesterol, *HDL-C* high-density lipoprotein cholesterol, *LDL-C* low-density lipoprotein cholesterol, *TG* triglyceride, *UA* uric acid, *FPG* fasting plasma glucose, *HOMA-IR* homeostasis model assessment insulin resistance, *Log-transformed variables for non-normally distributed variables (AST, ALT, γ-GTP, TG, FPG, insulin, HOMA-IR) were used to analysis. ^†^The low-density lipoprotein cholesterol (LDL-C) was calculated using the Friedewald equation. ^‡^ *P* value for difference between heavyweight and nonheavyweight groups was assessed by unpaired *t* test. Significant differences, *P* < 0.05

**Table 4:** Comparison of blood biochemical parameters between heavyweight Judo and football groups

	Heavyweight (*n* = 41)		*P* value^‡^	Normal range
	Judo (*n* = 19)	Football (*n* = 22)		
AST* (U/L)	28 (20–44)	30 (24–37)	0.856	10–40
ALT* (U/L)	36 (23–54)	40 (31–48)	0.872	5–45
γ-GTP* (U/L)	29 (23–43)	35 (28–47)	0.371	<80
Total-C (mg/dL)	174 ± 33	183 ± 31	0.373	120–219
HDL-C (mg/dL)	47 ± 8	49 ± 8	0.470	40–85
LDL-C^†^ (mg/dL)	96 ± 35	112 ± 24	0.107	≦140
TG* (mg/dL)	135 (90–185)	103 (66–131)	0.107	30–149
UA (mg/dL)	7.4 ± 1.3	6.4 ± 1.5	0.026	3.8–7.0
FPG* (mg/dL)	83 (79–90)	87 (83–91)	0.333	70–109
Insulin* (μIU/mL)	8.6 (4.9–11.7)	7.7 (5.8–11.4)	0.707	1.7–10.4
HOMA-IR*	1.99 (1.05–2.42)	1.58 (1.16–2.77)	0.631	<2.50

Data were expressed as mean ± SD or median (inter quartile range). *AST* aspartate aminotransferase, *ALT* alanine aminotransferase, *γ-GTP* γ- glutamyl transpeptidase, *Total-C* total cholesterol, *HDL-C* high-density lipoprotein cholesterol, *LDL-C* low-density lipoprotein cholesterol, *TG* triglyceride, *UA* uric acid, *FPG* fasting plasma glucose, *HOMA-IR* homeostasis model assessment insulin resistance; *Log-transformed variables for non-normally distributed variables (AST, ALT, γ-GTP, TG, FPG, insulin, HOMA-IR) were used to analysis. ^†^The low-density lipoprotein cholesterol (LDL-C) was calculated using the Friedewald equation. ^‡^ *P* value for difference between heavyweight Judo and Football groups were assessed by unpaired *t* test. Significant differences, *P* < 0.05

## Discussion

This study examined the characteristics of body composition and cardiometabolic risk in heavyweight Judo athletes compared with heavyweight athletes in other sports. Our main finding in this study was that although heavyweight Judo athletes had higher body fat percentage and more fat mass, especially VF, their cardiometabolic risk in terms of blood biochemical parameters was not significantly higher than that in heavyweight football athletes. Mean BM of the heavyweight Judo group was approximately 20 kg heavier, and mean body fat percentage was approximately 8 % higher than those of the heavyweight football group. Additionally, VF area of the heavyweight Judo group was about two times larger than that of the heavyweight football group (Table 2). Previous studies of non-athletes showed that cardiovascular disease was associated with accumulation of excess VF [11, 12]. Therefore, we expected that the heavyweight Judo group would have higher cardiometabolic risk compared with the heavyweight football group. However, most blood biochemical variables were not significantly different between both heavyweight groups, except for UA concentration (Table 4). It is difficult to explain why the large fat mass of the heavyweight Judo group did not increase their cardiometabolic risk such as lipid profile and insulin relative to the heavyweight football group from results of this study. However, simply having more fat mass may not necessarily increase cardiometabolic risk in athletes. Previous studies reported the existence of metabolically healthy obese individuals, who have low risk of cardiovascular disease and insulin resistance [13, 14]. For example, a follow-up study of metabolically healthy obese Italian individuals showed no increase in cardiovascular disease risk over 15 years [15]. Although the mechanism of metabolically healthy obesity is not clearly understood, it has been reported that increased physical activity may offset the high risk of cardiovascular disease in these individuals [16]. Additionally, another previous study reported that a high level of cardiorespiratory fitness was associated with lower prevalence of metabolic risk, even among obese individuals [17]. The heavyweight Judo athletes in this study participated in daily training for approximately 3 h including not only strength exercises but also a lot of high-intensity intermittent exercises which require physiological endurance. In fact, high-intensity intermittent exercise is a specific modality of Judo training [18]. Such training programs may help to increase their physical activity level and cardiorespiratory fitness regardless of their heavy BM [19, 20]. Previous study demonstrated that male heavyweight Judo athletes in elite team have comparable aerobic capacity to professional defensive linemen as heavyweight football athletes [21, 22]. Therefore, Judo athletes may be similarly metabolically active as heavyweight football players, and simply having more fat mass does not automatically increase cardiometabolic risk for athletes. At the same time, those heavyweight athletes still have higher risk of cardiometabolic risk compared to nonheavyweight athletes. Therefore, periodic biochemical tests to assess such risks are suggested to detect heavyweight athletes at high cardiometabolic risk. We should also note that only UA concentration was higher in the heavyweight Judo group compared with in the heavyweight football group (Table 4). The high concentration of UA is known to cause gout, and it has been reported that VF is the most influential factor in UA concentration [23]. Therefore, the larger VF mass of the heavyweight Judo athletes may have increased their UA concentration. At the same time, it has been reported that UA metabolism is influenced by both dietary intake and physical exercise [24]. High intake of meat and fish is known to be the dietary cause of increase in blood UA level. Heavyweight Judo athletes in this study may have a high intake of meat products to increase their energy and protein intake to gain BM as muscle mass. Moreover, high-intensity intermittent exercise increase hypoxanthine and UA metabolism [25]. Therefore, it is possible that diet and exercise may influence the UA level of heavyweight Judo athletes besides their large VF mass. However, diet and exercise were not assessed in this study. Therefore, further study to evaluate the influence of diet and/or exercise on cardiometabolic risk, including UA concentration, will help to elucidate the development of risk in heavyweight athletes.

The prevalence of abnormally high UA and TG levels was higher in the heavyweight Judo group compared with those in the nonheavyweight group (Fig. 1), even though mean UA and TG levels in the heavyweight Judo group were within or close to the reference normal range (Table 4). In other words, there are several heavyweight Judo athletes in this study who had abnormally high levels of UA and/or TG, but most of the heavyweight athletes were within its normal ranges by observing the individual results. Those athletes with higher UA and/or TG level did not necessarily carry more fat mass or VF mass. Additionally, there were no other anthropometric parameters commonly found in those who had abnormal blood biochemical parameters among Judo athletes. Therefore, athletes with increased cardiometabolic risk may be influenced by other factors such as genetic factors [26, 27], dietary habits [28, 29], or other lifestyle factors commonly related with cardiometabolic risks.

The present study has a few limitations. Firstly, this study design was a cross-sectional study, and thus, it is difficult to determine the cause and effect relationship in this study. Furthermore, larger sample sizes, prospective and interventional studies, are required to investigate the physiological mechanism of decreasing cardiometabolic risk in heavyweight Judo athletes despite their excessive fat mass.

## Conclusion

Cardiometabolic risk in Japanese male heavyweight Judo athletes was not significantly higher compared with other heavyweight athletes, even though they had higher fat mass such as VF. Therefore, excessive fat accumulation may not necessarily increase cardiometabolic risk for heavyweight Judo athletes.

## Abbreviations

ALT: alanine aminotransferase
AST: aspartate aminotransferase
BM: body mass
BMI: body mass index
DXA: dual-energy X-ray absorptiometry
FFM: fat-free mass
HDL-C: high-density lipoprotein cholesterol
HOMA-IR: homeostasis model assessment insulin resistance
LDL-C: low-density lipoprotein cholesterol
MRI: magnetic resonance imaging
SF: subcutaneous fat
TG: triglyceride
UA: uric acid
VF: visceral fat
γ-GTP: γ-glutamyl transpeptidase
