# Erratum to: ‘Characteristics of body composition and cardiometabolic risk of Japanese male heavyweight Judo athletes’

**DOI:** 10.1186/s40101-016-0093-7

**Published:** 2016-04-19

**Authors:** Hiroko Murata, Satomi Oshima, Suguru Torii, Motoko Taguchi, Mitsuru Higuchi

**Affiliations:** 1 Graduate school of Sport Sciences, Waseda University, 112 Frontier Reseach Center 135-1 Horinouchi, Tokorozawa-shi, Saitama 359-1192 Japan; 2 Faculty of Sport Sciences, Waseda University, 2-579-15 Mikajima, Tokorozawa-shi, Saitama 359-1192 Japan

Unfortunately, the original version of this article [1] contained an error. The caption for Fig. 1 was included incorrectly. The correct caption can now be found below.

Fig. 1 Comparison of prevalence in cardiometabolic risk in terms of blood biochemical parameters. Prevalence of cardiometabolic risk presents the percentage over the referenced normal range of respective parameters. The cut-off referenced normal range were as follows: AST > 40 (U/L), ALT > 45 (U/L), γ-GTP ≧80 (U/L), HDL-C < 40 (mg/dL), LDL-C > 140 (mg/dL), TG ≧150 (mg/dL), UA > 7.0 (mg/dL), HOMA-IR ≧2.5. *Significant differences compared with Nonheavyweight group, *p* < 0.05.

## References

[1] Murata H, Oshima S, Torii S, Taguchi M, Higuchi M (2016). Characteristics of body composition and cardiometabolic risk of Japanese male heavyweight Judo athletes. J Physiol Anthropol.

